# Flexible Polycaprolactone and Polycaprolactone/Graphene Scaffolds for Tissue Engineering

**DOI:** 10.3390/ma12182991

**Published:** 2019-09-16

**Authors:** Stanislav Evlashin, Pavel Dyakonov, Mikhail Tarkhov, Sarkis Dagesyan, Sergey Rodionov, Anastasia Shpichka, Mikhail Kostenko, Stepan Konev, Ivan Sergeichev, Petr Timashev, Iskander Akhatov

**Affiliations:** 1 Center for Design Manufacturing & Materials, Skolkovo Institute of Science and Technology, Bolshoy Boulevard 30, bld. 1, 121205 Moscow, Russia; S.Evlashin@skoltech.ru (S.E.); s.konev@skoltech.ru (S.K.); i.sergeichev@skoltech.ru (I.S.); i.akhatov@skoltech.ru (I.A.); 2 Skobeltsyn Institute of Nuclear Physics, Lomonosov Moscow State University, 1(2) Leninskiye Gory, 119991 Moscow, Russia; 3 Institute of Nanotechnologies of Microelectronics of the Russian Academy of Sciences, 32 A Leninsky Prospekt, 119991 Moscow, Russia; tmafuz@mail.ru; 4 Faculty of Physics, Lomonosov Moscow State University, 1-2 Leninskie Gory, 119991 Moscow, Russia; capkuckokos@gmail.com; 5 N. N. Priorov National Medical Research Center of Traumatology and Orthopaedics, 10 Priorov, 127299 Moscow, Russia; rodionov_085@mail.ru; 6 Institute for Regenerative Medicine, Sechenov First Moscow State Medical University, 8-2 Trubetskaya st., 119991 Moscow, Russia; ana-shpichka@yandex.ru (A.S.); timashev.peter@gmail.com (P.T.); 7 Kurnakov Institute of General and Inorganic Chemistry of Russian Academy of Sciences, 31 Leninskiy Prospect, 119071 Moscow, Russia; kostenko@supercritical.ru

**Keywords:** scaffold, graphene oxide, polycaprolactone, supercritical foaming, flexible composite

## Abstract

Developing bone scaffolds can greatly improve the patient’s quality of life by accelerating the rehabilitation process. In this paper, we studied the process of composite polycaprolactone supercritical foaming for tissue engineering. The influence of graphene oxide and reduced graphene oxide on the foaming parameters was studied. The structural and mechanical properties were studied. The scaffolds demonstrated mechanical flexibility and endurance. The co-culturing and live/dead tests demonstrated that the obtained scaffolds are biocompatible. Different composite scaffolds induced various surface cell behaviors. The experimental data demonstrate that composite foams are promising candidates for in vivo medical trials.

## 1. Introduction

Large bone defects lead to a significant decrease in a patient’s quality of life. Such trauma requires significant rehabilitation time. To reduce the rehabilitation period, different artificial scaffolds are being developed [1]. Scaffolds for bone regeneration should meet certain requirements: biocompatibility, biodegradability and mimicking the structural and mechanical properties of bone [2]. Additionally, scaffolds need to induce osteoprogenitor cell migration, proliferation, and differentiation, start the formation of new cell matrix and support vasculature growth and development through the body of the scaffold [3]. Currently, synthetic (Poly(lactic-coglycolic acid) (PLGA), Polycaprolactone (PCL), Phenylmagnesium chloride (pHMGCL), poly-lactic-co-glycolic acid–polyethylene oxide (PLGA-PEO), etc.) and natural (gelatin/chitooligosaccharide, collagen, chitosan, etc.) polymers and composites are used for scaffold fabrication [4].

Graphene, graphene oxide (GO) and reduced graphene oxide (rGO) are potential candidates for implementation in tissue engineering applications [5,6]. Some scientific results have demonstrated that graphene incorporation improves mechanical properties, cell adhesion and proliferation [7,8,9]. Recently, additive technologies were applied to fabricate sophisticated shapes of graphene/polymer materials. A number of studies have demonstrated on-demand scaffold printing of various shapes from composite materials [10,11]. The main benefit of this approach is the complexity of the structures. A composite graphene polymer scaffold was printed and used not only for bone injury but also for nerve regeneration [12].

An alternative approach for the fabrication of composite GO/polymer materials is the preparation of electrospun mats [13]. This technique is mostly studied for polymers such as PCL and PLGA [14].

One alternative 3D scaffold fabrication approach is supercritical foaming in a carbon dioxide atmosphere [15,16]. This technique allows a certain level of control over the porosity and cell morphology of the scaffold. Supercritical foaming can be implemented for polymer or polymer/graphene scaffold fabrication [17,18]. In these papers, the fabrication of poly(lactic acid)/graphene and polystyrene/GO composites of different structures and porosities were studied.

Although many scientific papers have focused on the biomedical activity of graphene derivatives, none has led to clinical trials, and there is yet no distinct understanding of the effects of graphene derivatives on humans [19]. The properties of graphene-based materials strongly depend on the synthesis process because a wide variety of resulting material properties have been reported by different groups using the same synthesis approach [19].

In this work, we studied the process of supercritical foaming of polycaprolactone and polycaprolactone/graphene composite in a carbon dioxide atmosphere. We investigated the dependence of foaming conditions on the mechanical and structural properties of polycaprolactone scaffolds and composite polycaprolactone/graphene foam. For the first time such mechanical properties as mechanical stability (fatigue tests) of PCL and PCL/rGO scaffolds were investigated. The biocompatibility of the obtained porous structures was also studied.

## 2. Materials and Methods

### 2.1. Materials

Polycaprolactone (Sigma Aldrich, St. Louis, MO, USA) with a molar mass of M_n_ = 80 kDa was used for sample fabrication. GO was prepared from Timrex KS 15 graphite powder (Timcal Ltd, Bodio, Switzerland) as demonstrated in our earlier work [20,21]. Chimmed high purity acetone was used as a mutual solvent for the polymer and GO.

### 2.2. Composite Material Production

The level of reduction for graphene oxide is determined by the amount of oxygen-containing groups present on the surface of graphene sheets. At the same time, visual color of the material is different, GO is brown while rGO is black. Materials used in this study were both prepared of the same suspension and were reduced due to presence of heat treatment during fabrication process. However one of these material faced less treatment, and remained “brown” and is called GO, there other one was more exposed to heat treatment, thus leaving less oxygen percentage on the surface and is called rGO. The color of this sample turned black.

GO was produced with a standard Hummers method [22]. Two different approaches were used to fabricate the composite graphene/polymer. In the first approach, we used an aqueous solution of GO, which was further subjected to a centrifugal process to divide water from GO and repeatedly substitute it with acetone to remove all the water traces. Then, polycaprolactone was added to the acetone/GO solution and thoroughly mixed, and then a temperature of 80 °C was applied to dry out the acetone. During the drying stage, GO was reduced due to the heat treatment [23]. A composite material with a uniform distribution of reduced GO was obtained as a result of this procedure. This material will be called PCL/GO.

The second method is based on the hydrothermal reduction of GO, which leads to the formation of graphene foam [24]. After fabrication of the porous graphene structure, water was replaced by acetone 10 times repeatedly. Then, the graphene foam was fractured and dispersed in acetone using an ultrasonication tip (Unicorn system MEPH93.1 MELPHYS, Moscow, Russia) for 15 min. The suspension was mixed with polycaprolactone and dried at 80 °C. This material will be called PCL/rGO.

As a final step, all obtained materials were dried in a vacuum at 120 °C for 8 h to remove all of the residual acetone.

### 2.3. Scaffold Fabrication

We used supercritical foaming in a carbon dioxide atmosphere for scaffold fabrication. Experimental set up of CO_2_ pump, chamber with a pressure reduction vessel, thermocouple-sensing and pressure-sensing elements was used. A detailed description is listed in [16]. This set up allows pressures up to 300 atm and temperatures up to 80 °C to be reached. The morphology of the structures was controlled by changing the decompression rate.

All experimental conditions were fixed except for the decompression rate. The chamber pressure and foaming time were 180 atm and 60 min, respectively.

### 2.4. Chemical Structure and Morphology Analysis

Morphological analysis of the obtained materials was performed with a Axio Scope A1 microscope (Carl Zeiss, Oberkochen, Germany) and a Carl Zeiss Supra 40 SEM (Carl Zeiss, Oberkochen, Germany). For analysis of chemical structure, we used Bruker 70v FTIR (Bruker, Billerica, MA, USA) and DXR Raman microscope spectrometers (Thermo Scientific, Waltham, MA, USA). For mass and freezing/melting point analysis, the DSC 60 Plus (Shimadzu, Kyoto, Japan) and DTG 60 (Shimadzu, Kyoto, Japan) systems were used. The mechanical properties were studied with an ElectroPuls E3000 (Instron, Norwood, MA, USA) and Digital Image Correlation (DIC) VIC-3D System (Correlated Solutions, Irmo, SC, USA). A PB1000 (Nanovea, Irvine, CA, USA) mechanical tester was used for hardness analysis.

### 2.5. Biocompatibility

Biocompatibility tests were performed via co-culturing the scaffolds with rabbit marrow stromal cells. The stromal cells were obtained by mechanical fragmentation of an iliac crest piece with subsequent disaggregation in a 0.2% solution of type I collagenase (PanEco, Moscow, Russia) over a 4-h period. The obtained solution was filtered with a 40 μm cell bolster. Collagenase was removed from the effluents by centrifugation (200 g, 10 min). The medium was renewed every 3 days. The culture was passaged with a 0.25% trypsin and versene solution (PanEco).

During the second passage, stage cell co-culturing with a fragmented scaffold material with 10^5^ cells per square centimeter was performed. The co-culturing duration was 2 weeks. After that period, the cells were dyed for the LIVE/DEAD test (Thermo Scientific, Waltham, MA, USA) as per the manufacturer’s recommended procedure. The dyed samples were studied with a laser confocal scanning microscope (Ti-E, Nikon, Minato, Tokyo, Japan) followed by image processing using NIS-Elements software (Nikon Instruments Inc., Melville, NY, USA).

The cytotoxicity of scaffolds was assessed via Lactate DeHydrogenase (LDH) (Thermo Scientific, Waltham, MA, USA) and 3-(4,5-Dimethylthiazol-2-yl)-2,5-diphenyltetrazolium bromidefor (MTT) (Sigma Aldrich St., Louis, MO, USA) assays using MSC culture in accordance with the manufacturer’s instructions. Extracts from scaffolds were prepared at a concentration of 1.2 mg/mL (DMEM/F12 with 5% FBS and 1% penicillin-streptomycin) as described in international measurement standard ISO 10993-12:2012 and [25]. We seeded 5000 cells per well and added extracts in 24 h. In MTT assay, the five extract dilutions were used (six concentrations in total). In LDH assay, cell lysate was used as a positive control for the maximum LDH release, and the cell culture treated with water – as a negative control for the minimum (spontaneous) LDH release. In MTT assay, sodium dodecyl sulphate dilutions were applied as a positive control. The absorbance spectra were measured at 492 nm (LDH assay) and 570 nm (MTT assay) using a microplate reader Victor Nivo (PerkinElmer, Waltham, MA, USA); 680 nm and 650 nm, respectively, were used as reference wavelength. The results validity was ensured by the analysis of triplicate experiments; each data point represents the mean standard deviation. The analysis was carried out using ANOVA (Statistics Solutions, Clearwater, FL, USA); results with *p*-value <0.05 were considered as statistically significant.

## 3. Result and Discussion

**Figure 1:**
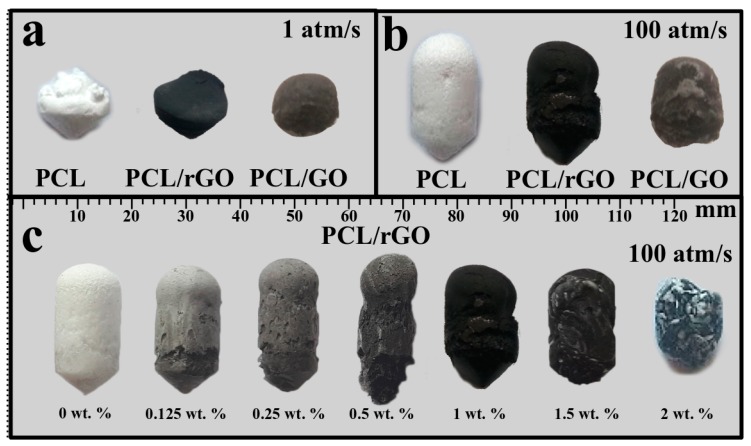
PCL, PCL/GO, PCL/rGO scaffolds produced at decompression rates of (**a**) 1 atm/s and (**b**) 100 atm/s and (**c**) PCL/rGO scaffolds filled with different rGO concentrations.

Similar to the results of other polymer-foaming processes, an increase in decompression rate for PCL, PCL/GO and PCL/rGO led to an increase in the general size of the foam, thus leading to an increase in the pore size (Figure 1 a,b).

Figure 1a,b demonstrate scaffolds of the same materials prepared at two different pressure regimes. Figure 1c demonstrates scaffolds synthesized under the same conditions but with different rGO concentrations. When the rGO content increases, the PCL/rGO mixture stops being uniform, and the scaffold size decreases. The concentration limit at which the scaffold mixture remains uniform is 1.5 wt. %, and a further increase in the GO/rGO content causes the scaffold structure to be uneven, thus leading to the presence of regions with distinct properties, which tend to foam in a different fashion. Because plastification and nucleation occur faster in raw PCL, the PCL/graphene composites with rGO contents greater than 2 wt. % tend to have white regions of PCL, due to that fact, this concentration will be the maximum concentration considered.

The increase in temperature to 80 °C and the foaming time increase to 4 h had no significant impact on the scaffold structure and did not lead to a uniform distribution of rGO.

The median value of 1 wt. % concentration was chosen for SEM investigation, since 0 wt. % was already studied in other scientific works and 2 wt. % possesses no interest due to absence of uniform distribution.

**Figure 2:**
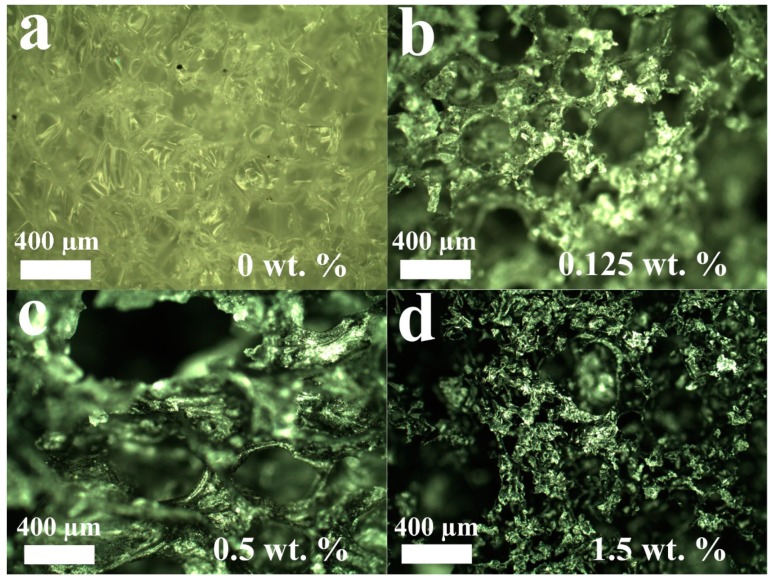
Optical images of PCL/rGO scaffolds. (**a**–**d**) demonstrate different rGO concentrations.

**Figure 3:**
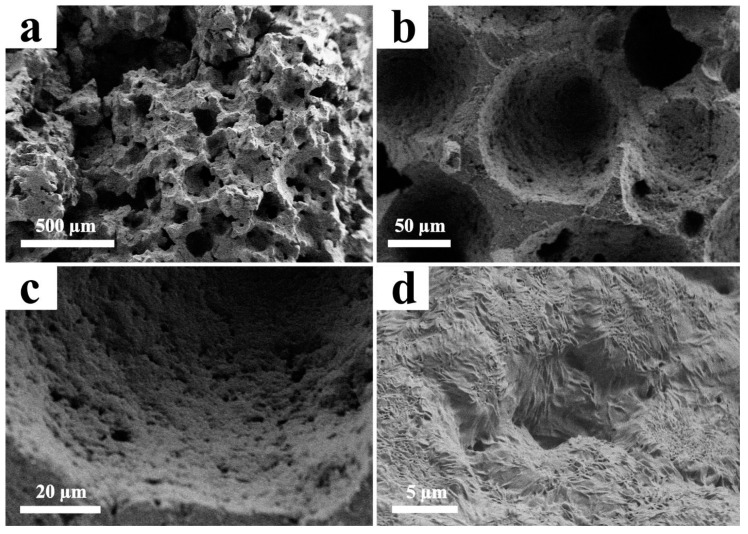
SEM images of PCL/rGO produced at 180 atm. (**a**–**d**) demonstrate different magnifications of scaffold structure. The time in the supercritical medium is 60 min, the decompression rate is 100 atm/s, and the rGO concentration is 1 wt. %.

Figure 2 demonstrates optical images of PCL/rGO scaffold porous structure. As it can be observed, increase in rGO content leads to formation of pores of higher average volume. The SEM images are presented in Figure 3.

Data on pore size was collected from several SEM images for estimation of average pore size. An estimated pore size of 100–200 μm was obtained from the SEM images; however, some pores of several microns can be observed in the enhanced images. A surface with this morphology is suitable for cell proliferation [26].

**Figure 4:**
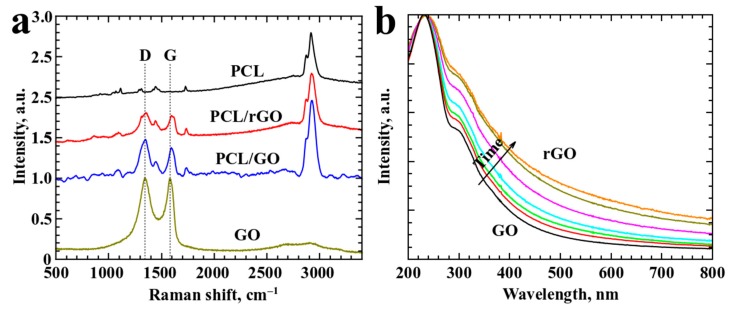
(**a**) Raman spectra of samples and (**b**) absorption spectra of GO solutions after different thermal treatment times (t_max_ = 8 h, 80 °C).

Raman analysis provided the presence of standard D and G carbon bands in the spectra of all samples with GO and rGO content (see Figure 4a) [27,28]. FTIR analysis did not provide any significant results.

Absorption spectra (Figure 4b) shows data for GO samples exposed to various times of heat treatment in order to obtain samples of various levels of reduction. GO absorption spectrum demonstrates characteristic bands at ~235 nm and ~300 nm, which tend to shift towards red part of the spectrum upon increase of reduction level. Heat treatment induces decrease in band intensity and slight redshift typical for process of rGO reduction indicating removal of some oxygen groups [29]. Removal of some amount of oxygen groups leads to transformation of sp^2^ into sp^3^ bonding, thus increasing optical absorption of the material [30].

To investigate temperature influence on the suspension of GO, additional tests were conducted. The suspension of GO was placed in an oven at 80 °C for 8 h. The absorption spectrum undergoes considerable changes, which indicates reduction occurring in GO [23]. During the drying process, the samples are placed in a vacuum dryer at 120 °C; thus, reduction occurs faster at higher temperatures.

**Figure 5:**
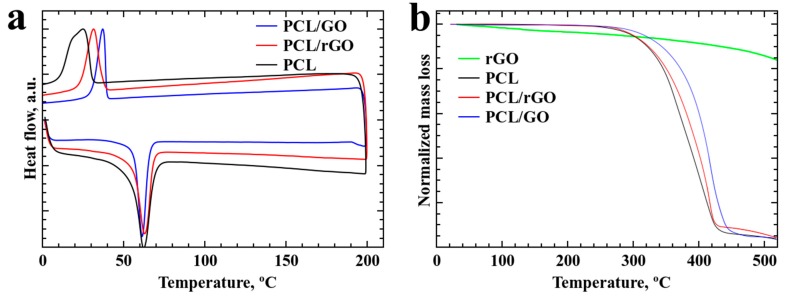
(**a**) DSC and (**b**) TGA spectra of the samples.

The DSC and TGA spectra are presented in Figure 5. Figure 5a shows that melting occurs at the same point for all composite materials; however, samples with GO demonstrate higher crystallization temperatures, which can be explained by the uniform mixture of polymer and GO. PCL/rGO also demonstrates an increased crystallization temperature, yet lower than that of PCL/GO. The temperature shift is more than 10 °C: 25 °C for PCL, 32 °C for PCL/rGO, and 37 °C for PCL/GO.

The same tendency was observed in the TGA results of the samples. The PCL/GO samples demonstrate the highest decomposition temperature, which can also be explained by the uniform mixture of polymer and GO.

Mechanical characteristics were studied for the obtained samples. Graphene addition leads to an increase in sample hardness and a significant decrease in elongation. The hardness values of solid PCL, 1% PCL/rGO and PCL/GO composites are 4.8, 7.6 and 7.9 HV, respectively. The Young’s modulus values are 365 MPa, 700 MPa and 1500 MPa, respectively. 

**Figure 6:**
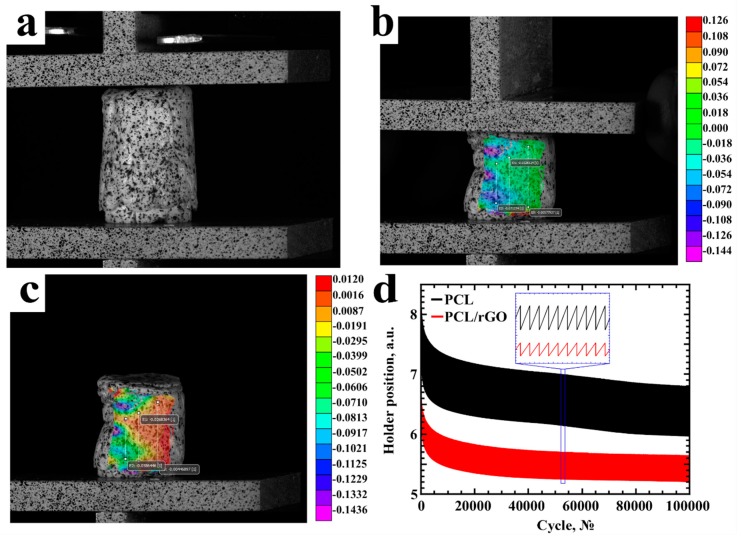
Mechanical studies of PCL samples with loads from 20 to 50 N. (**a**) Sample without loading; (**b**) stress field, obtained with use of DIC (Digital Image Correlation) during the mechanical tests; (**c**) DIC system loading after the mechanical measurements; (**d**) fatigue tests (inset shows the close up of the cycling compression test curve).

The produced scaffolds will be used for tissue engineering applications, such as osteo- and chondro-scaffolding, which require repetitive mechanical loading. The fatigue tests are presented in Figure 6. The PCL (Figure 6a–c) and PCL/rGO samples were tested with loads of 20–50 N. The PCL/GO samples demonstrated brittle behavior even at preparatory phase of fatigue tests, due to the fact that no loading could have been applied without destroying the sample.

According to the results of fatigue tests (Figure 6d) we concluded that the PCL and PCL/rGO samples are flexible and can undergo repetitive loadings for more than 100,000 loading cycles. Moreover, for the same size of scaffolds, the same loads lead to different deformations. The deformation of PCL/rGO at the same loading is less than that of raw PCL (Figure 6 inset).

**Figure 7:**
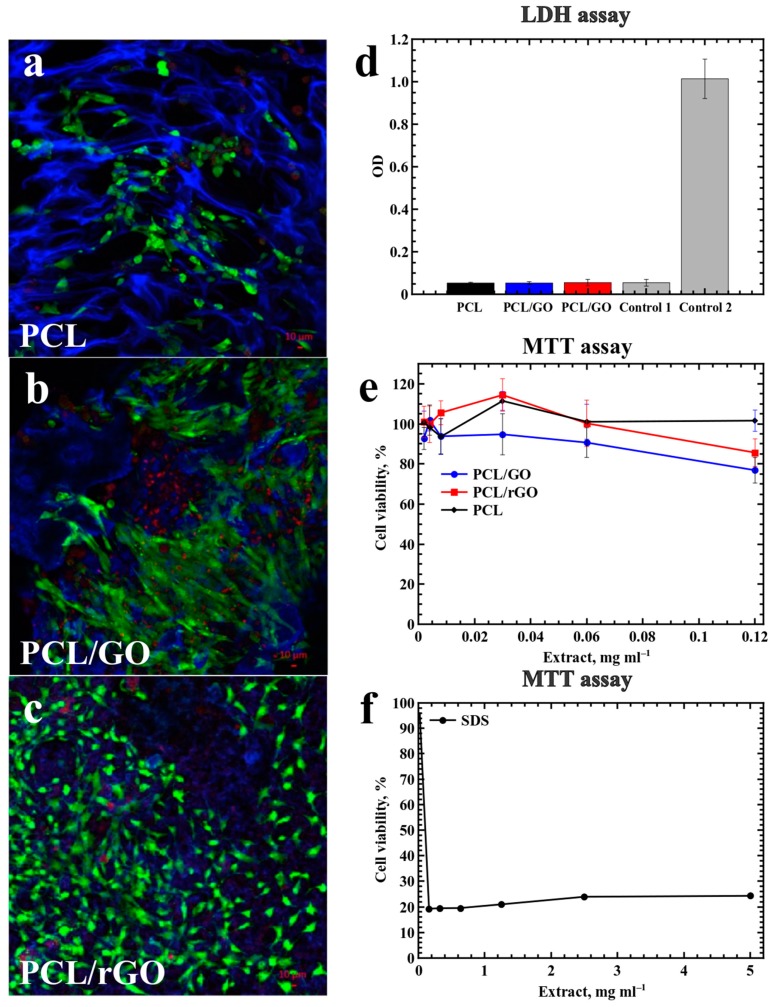
Cytotoxicity of PCL, PCL/GO, and PCL/rGO scaffolds. (**a**–**c**) Live/Dead staining (live cells—green (Calcein AM), dead cells—red (propidium iodide), scaffold—blue), laser scanning confocal microscopy. (**d**) lactate dehydrogenase release (LDH assay): Control 1—spontaneous LDH release as a negative control; Control 2—maximum LDH release (cell lysate) as a positive control. (**e**,**f**) Cell viability (MTT assay): Sodium Dodecyl Sulfate (SDS) as a positive control.

PCL, PCL/GO and PCL/rGO scaffolds were tested for cytotoxicity using LIVE/DEAD staining and LDH and MTT-assays (Figure 7).

The PCL, PCL/GO, PCL/rGO samples with 1% of GO content were chosen for the biocompatibility studies. The biocompatibility estimation of cells co-cultured with the PCL, PCL/GO and PCL/rGO scaffolds demonstrated that cells remained viable. When fragmented material was placed into a flask with a cell monolayer, the cells did not detach and were able to proliferate.

When the cells were applied to the substrate surface, the cells attached to it and spread across, keeping the capacity to proliferate up to 14 days (Figure 7a–c). Most cells remained fusiform or triangular fibroblastic in shape, an intrinsic property for cells in monolayer culture. Interestingly, cells on the surface of the PCL/GO sample tended to form short sharp branches, forming a branched shape.

Cell adhesion to the scaffold material and subsequent cell spreading show potential for cell migration on the substrate surface. The adhesion of the cell to the substrate varies. On the surfaces of PCL and PCL/rGO, the cells were spherical, demonstrating poor adhesion compared to that of cells on PCL/GO (Figure 7a,c). The branch appearance on the PCL/GO sample can be explained by the interaction between the scaffold surface features and cells (Figure 7b).

The absence of cells inside the scaffold structure indicates the inability of the cells to penetrate the foam due to the non-interconnected morphology of the pores in all three types of materials. The hollow structures inside the scaffolds are encapsulated. On the one hand, these pores do not promote smooth cell migration across the scaffold structure or free development of vasculature through the scaffold volume. However, on the other hand, these pores can be filled with antibiotics, which can be released during the biodegradation of the scaffold [11].

The results of the quantitative cytotoxicity assessment showed that the analyzed scaffolds did not possess significant toxicity: LDH release in samples coincided with the spontaneous release, and the cell viability for all sample dilutions was higher than 70% (Figure 7d,e). However, the lowest cell viability was revealed for PCL/GO scaffolds 76.8 ± 6.4.

This study demonstrated that PCL, PCL/GO and PCL/rGO scaffolds are biocompatible; thus, these structures can potentially be tested for in vivo biodegradation.

## 4. Conclusions

In this work, we demonstrate a simple method of scaffold production from PCL, PCL/GO and PCL/rGO. The scaffolds were produced using supercritical foaming in a carbon dioxide atmosphere. The composition and structural properties of the obtained materials were studied with SEM, DSC, TGA, Raman and FTIR analysis. We estimated the maximum concentration of GO and rGO to be ~2 wt. %. Further increases in the GO and rGO contents led to the formation of non-homogenous scaffolds. A digital image correlation system and fatigue test were used to study the mechanical properties. The PCL and PCL/rGO foams demonstrated good flexibility and capability to undergo 10^5^ loading cycles. The machine for mechanical measurements was set for 10^5^ number of cycles and we believe that tested samples possess potential to undergo even a larger amount of loading cycles. However, the PCL/GO composites did not show flexibility and were destroyed under external loading. All materials demonstrated biocompatibility properties. Cell adhesion to the PCL/rGO scaffold was better than that to the PCL and PCL/GO scaffolds. The developed material possesses the potential for bone implant applications.

## References

[1] Petite H., Viateau V., Bensaïd W., Meunier A., de Pollak C., Bourguignon M., Oudina K., Sedel L., Guillemin G. (2000). Tissue-engineered bone regeneration. Nat. Biotechnol.

[2] Roseti L., Parisi V., Petretta M., Cavallo C., Desando G., Bartolotti I., Grigolo B. (2017). Scaffolds for bone tissue engineering: State of the art and new perspectives. Mater. Sci. Eng. C.

[3] Dong L., Wang S.-J., Zhao X.-R., Zhu Y.-F., Yu J.-K. (2017). 3D- printed poly(ε-caprolactone) scaffold integrated with cell-laden chitosan hydrogels for bone tissue engineering. Sci. Rep..

[4] Polo-Corrales L., Latorre-Esteves M., Ramirez-Vick J.E. (2014). Scaffold design for bone regeneration. J. Nanosci. Nanotechnol.

[5] Chung C., Kim Y.-K., Shin D., Ryoo S.-R., Hong B.H., Min D.-H. (2013). Biomedical applications of graphene and graphene oxide. Acc. Chem. Res..

[6] Holt B.D., Wright Z.M., Arnold A.M., Sydlik S.A. (2017). Graphene oxide as a scaffold for bone regeneration. Wiley Interdiscip. Rev. Nanomed. Nanobiotechnol..

[7] Song J., Gao H., Zhu G., Cao X., Shi X., Wang Y. (2015). The preparation and characterization of polycaprolactone/graphene oxide biocomposite nanofiber scaffolds and their application for directing cell behaviors. Carbonn. Y.

[8] Chartarrayawadee W., Molloy R., Ratchawet A., Janmee N., Butsamran M., Panpai K. (2017). Fabrication of poly(lactic acid)/graphene oxide/stearic acid composites with improved tensile strength. Polym. Compos..

[9] Sayyar S., Murray E., Thompson B.C., Gambhir S., Officer D.L., Wallace G.G. (2013). Covalently linked biocompatible graphene/polycaprolactone composites for tissue engineering. Carbonn. Y.

[10] Zhou X., Nowicki M., Cui H., Zhu W., Fang X., Miao S., Lee S.-J., Keidar M., Zhang L.G. (2017). 3D bioprinted graphene oxide-incorporated matrix for promoting chondrogenic differentiation of human bone marrow mesenchymal stem cells. Carbonn. Y.

[11] Sinha A., Choi Y., Nguyen M.H., Nguyen T.L., Choi S.W., Kim J. (2019). A 3D macroporous alginate graphene scaffold with an extremely slow release of a loaded cargo for in situ long-term activation of dendritic cells. Adv. Healthc. Mater..

[12] Jakus A.E., Secor E.B., Rutz A.L., Jordan S.W., Hersam M.C., Shah R.N. (2015). Three-dimensional printing of high-content graphene scaffolds for electronic and biomedical applications. Acs Nano.

[13] Jiang S., Chen Y., Duan G., Mei C., Greiner A., Agarwal S. (2018). Electrospun nanofiber reinforced composites: A review. Polym. Chem..

[14] Ege D., Kamali A.R., Boccaccini A.R. (2017). Graphene oxide/polymer-based biomaterials. Adv. Eng. Mater..

[15] Xu Z.-M., Jiang X.-L., Liu T., Hu G.-H., Zhao L., Zhu Z.-N., Yuan W.-K. (2007). Foaming of polypropylene with supercritical carbon dioxide. J. Supercrit. Fluids.

[16] Timashev P.S., Vorobieva N.N., Akovantseva A.A., Minaev N.V., Piskun Y.A., Kostjuk S.V., Selezneva I.I., Vasilenko I.V., Zakharkina O.L., Ignatieva N.Y. (2017). Biocompatibility and degradation of porous matrixes from lactide and ε-caprolactone copolymers formed in a supercritical carbon dioxide medium. Russ. J. Phys. Chem. B.

[17] Yang J., Wu M., Chen F., Fei Z., Zhong M. (2011). Preparation, characterization, and supercritical carbon dioxide foaming of polystyrene/graphene oxide composites. J. Supercrit. Fluids.

[18] Kuang T.-R., Mi H.-Y., Fu D.-J., Jing X., Chen B., Mou W.-J., Peng X.-F. (2015). Fabrication of poly(lactic acid)/graphene oxide foams with highly oriented and elongated cell structure via unidirectional foaming using supercritical carbon dioxide. Ind. Eng. Chem. Res..

[19] Kostarelos K., Novoselov K.S. (2014). Exploring the interface of graphene and biology. Science.

[20] Evlashin S.A., Svyakhovskiy S.E., Fedorov F.S., Mankelevich Y.A., Dyakonov P.V., Minaev N.V., Dagesyan S.A., Maslakov K.I., Khmelnitsky R.A., Suetin N.V. (2018). Ambient condition production of high quality reduced graphene oxide. Adv. Mater. Interfaces.

[21] Evlashin S., Dyakonov P., Khmelnitsky R., Dagesyan S., Klokov A., Sharkov A., Timashev P., Minaeva S., Maslakov K., Svyakhovskiy S. (2016). Controllable laser reduction of graphene oxide films for photoelectronic applications. Acs Appl. Mater. Interfaces.

[22] Hummers W.S., Offeman R.E. (1958). Preparation of graphitic oxide. J. Am. Chem. Soc..

[23] Kumar P.V., Bardhan N.M., Tongay S., Wu J., Belcher A.M., Grossman J.C. (2014). Scalable enhancement of graphene oxide properties by thermally driven phase transformation. Nat. Chem..

[24] Bosch-Navarro C., Coronado E., Martí-Gastaldo C., Sánchez-Royo J.F., Gómez M.G. (2012). Influence of the pH on the synthesis of reduced graphene oxide under hydrothermal conditions. Nanoscale.

[25] Shpichka A., Koroleva A., Kuznetsova D., Dmitriev R.I., Timashev P. (2017). Fabrication and handling of 3D Scaffolds Based on Polymers and Decellularized Tissues. Title of Advances in Experimental Medicine and Biology.

[26] Entezari A., Roohani I., Li G., Dunstan C.R., Rognon P., Li Q., Jiang X., Zreiqat H. (2018). Architectural design of 3d printed scaffolds controls the volume and functionality of newly formed bone. Adv. Healthc. Mater..

[27] Duan G., Fang H., Huang C., Jiang S., Hou H. (2018). Microstructures and mechanical properties of aligned electrospun carbon nanofibers from binary composites of polyacrylonitrile and polyamic acid. J. Mater. Sci..

[28] Zhou S., Zhou G., Jiang S., Fan P., Hou H. (2017). Flexible and refractory tantalum carbide-carbon electrospun nanofibers with high modulus and electric conductivity. Mater. Lett..

[29] Shi H., Wang C., Sun Z., Zhou Y., Jin K., Redfern S.A.T., Yang G. (2014). Tuning the nonlinear optical absorption of reduced graphene oxide by chemical reduction. Opt. Express.

[30] Eda G., Lin Y.-Y., Mattevi C., Yamaguchi H., Chen H.-A., Chen I.-S., Chen C.-W., Chhowalla M. (2010). Blue photoluminescence from chemically derived graphene oxide. Adv. Mater..

